# Primary Extraosseous Ewing Sarcoma of the Maxillary Sinus in an Adult-A Rare Case Report

**DOI:** 10.22038/ijorl.2019.35555.2173

**Published:** 2019-11

**Authors:** Ein-Wan Chin, Azreen-Zaira Abu-Bakar, Shahrul Hitam, Ngan Kah-Wai, Maizaton-Atmadini Abdullah

**Affiliations:** 1 Department of Otorhinolaryngology, Ampang Hospital, Selangor, Malaysia.; 2 Department of Pathology, Serdang Hospital, Serdang, Selangor, Malaysia.; 3 Department of Pathology, Faculty of Medicine and Health Sciences, University Putra Malaysia (UPM, Serdang, Selangor)

**Keywords:** Ewing, Epistaxis, Maxillary Sinus, Nasal Obstruction, Nasal Cavity, Sarcoma

## Abstract

**Introduction:**

Ewing sarcoma (ES), which is described as diffuse endothelioma of the bone, is divided into osseous and extraosseous Ewing sarcoma (EES) mostly affecting children and adolescents. It is a rare, aggressive, and poorly differentiated small blue round cell tumor that seldom affects the head and neck regions.

**Case Report:**

Herein, we reported a 46-year-old man presenting with right nasal block, epistaxis, and epiphora from the right eye for one month. The nasal endoscopy revealed a friable mass arising from the anterior half of the right nasal cavity. Histological findings were suggestive of Ewing sarcoma. A contrast-enhanced computed tomography (CT) scan of the paranasal sinuses showed a soft tissue mass in the right anterior nasal cavity with mucosal thickening in the right maxillary sinus, without any bony erosion or distant metastasis. The patient underwent endoscopic medial maxillectomy with modified Denker’s procedure, followed by a 6-cycle course of chemotherapy. He was clinically well after chemotherapy; however, the recent bone scans were suggestive of bone involvement with the tumor.

**Conclusion:**

The EES of paranasal sinus in the head and neck regions is extremely rare and requires exceptional attention due to their adjacent vital structures. The ES diagnosis-related dilemma arises from the numerous differential diagnoses of small round blue cell tumors. In this regard, accurate diagnosis is important, since ES requires a multi-modality approach. Furthermore, early diagnosis and aggressive intervention are crucial to obtain good prognosis and function.

## Introduction

Ewing sarcoma (ES) was first described as the diffuse endothelioma of bone by James Ewing in 1921(1). Angervall and Enzinger then introduced the term ‘extraosseous soft tissue ES’ in 1975 (2). 

The ES is divided into osseous/skeletal and extraosseous Ewing sarcoma (EES) that most often affects children and adolescents. The EES is a rare, aggressive, and poorly differentiated small blue round cell tumor, primarily in the soft tissues of the lower extremity and the paravertebral region. 

It seldom affects the head and neck region, accounting only for 1-4% of all ES (3). Herein, we reported a case of EES in order to highlight the clinical manifestations, diagnosis, imaging findings, and treatment of primary EES of maxillary sinus in an adult patient. 

## Case Report

**Fig 1:**
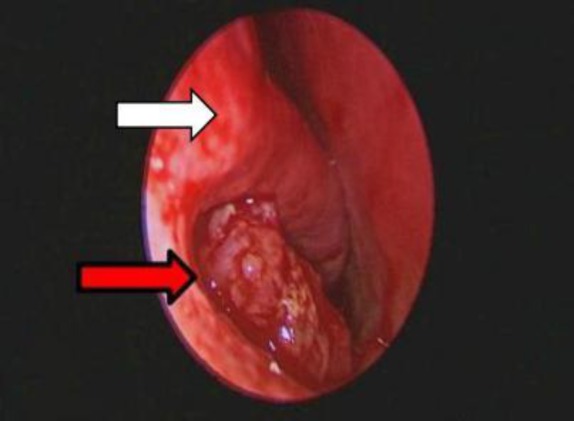
White arrow presenting inferior turbinate of right nasal cavity and red arrow showing an endoscopic view of tumor arising from right inferior meatus extending over anterior half of right nasal cavity

A 46-year-old Chinese man referred to us with one-month experience of gradually deteriorating right nasal block, epistaxis, and epiphora from the right eye. Nasoendoscopy revealed a reddish, friable mass arising from the right inferior meatus, extending over the anterior half of the right nasal cavity bleeding on touch (Fig.1). 

**Fig 2:**
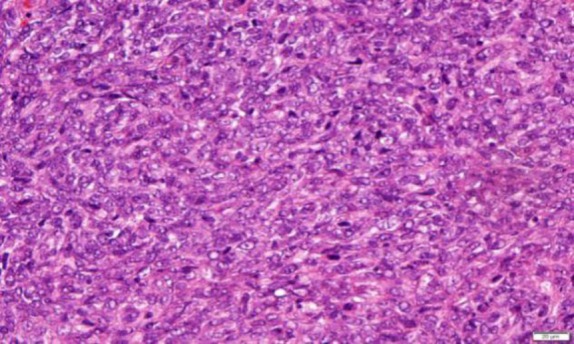
Diffuse infiltrates of small blue round cells with scanty cytoplasm and indistinct cell membrane (H&E stain)

The histology showed a malignant blue cell tumor infiltrating into the subepithelial stroma. Neoplastic cells displayed round to oval nuclei with fine chromatin and indistinct cytoplasmic membrane (Fig.2). 

A final diagnosis of ES/primitive neuroectodermal tumor (PNET) was made. The contrast-enhanced computed tomography (CT) scan showed a polypoidal soft tissue mass at the right anterior nasal cavity floor, measuring 0.7×1.0×0.7 cm with mucosal thickening observed in the right maxillary sinus, without any bony erosion and no evidence of distant metastasis (^Fig’s.3a,3b^). The patient underwent right endoscopic medial maxillectomy with modified Denker’s procedure.

**Fig 3:**
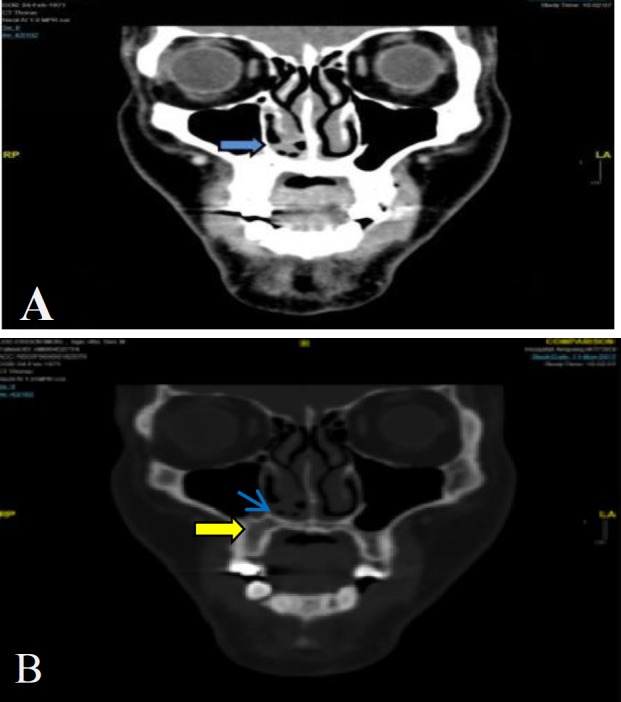
A) Contrast-enhanced computed tomography (CT) neck soft-tissue window coronal view of patient (blue arrow showing a soft tissue mass at the floor of right anterior nasal cavity), B) contrast-enhanced CT neck bone window coronal view of patient) yellow arrow showing the mucosal thickening in right maxillary sinus, blue arrow showing same soft tissue mass at the floor of right anterior nasal cavity, no bony erosion was seen adjacent to the tumor)

Intraoperative biopsy concurred with the initial biopsy diagnosis, and surgical margins were clear. He received three follow-up sessions over 3 months with monthly intervals. In the early stages, he complained of numbness and swelling on the right cheek, along with moderate epiphora; however, the conditions improved in subsequent follow-up. In this regard, no alar collapse was noted, and the rigid nasendoscopy showed good epithelialization at the operated site with no sign of relapse. Thereafter, he was referred to the oncology team with the intent of initiating postoperative chemotherapy, consisting of Vincristine, Ifosfamide, Doxorubicin, and Etoposide (VIDE regime), and completed six cycles. Repeated rigid nasendoscopy revealed no observed tumor relapse. After a month of post-chemotherapy, a bone scan was performed and revealed an increased tracer uptake over the right maxillary and nasal bone extending to the right orbital floor. 

This indicated sclerotic changes of the bone which were suggestive of bone involvement with tumor. In this regard, he was advised to refer to local radiation therapy; however, he refused to continue his treatment. He was last seen at our clinic 11 months post-operation, with no sign of local recurrence. He defaulted the follow-up since then. 

## Discussion

Involvement of the paranasal sinus (PNS) in the head and neck region EES is extremely rare, the majority of these cases were mentioned in the mandible, maxilla, maxillary sinus, ethmoid sinus and nasal cavity. It has a male to female ratio as high as 2.4:1, with a median age range of 11-20 years and prevalent among Caucasians (up to 95%). More than 90% of patients refer with a rapidly growing painful mass with the signs of early central nervous system extension. 

**Fig 4:**
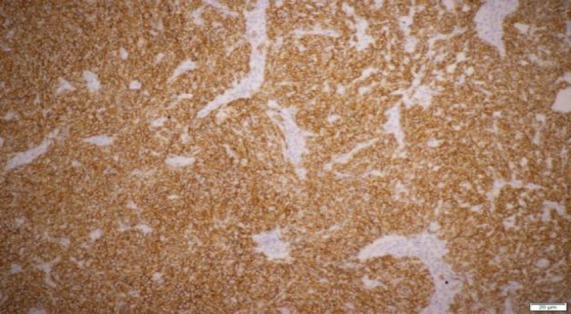
Tumour cells showing strong positivity for CD99 (immunohistochemical stain)

The tumor of the maxillary sinus presentation might be delayed until the lesion protrudes into the nasal or oral cavity and causes obvious obstructive or nasal symptoms (i.e., nose block or epistaxis), as presented in our patient. Other unspecific symptoms can be paraesthesia, ulceration, pyrexia, anemia, and weight loss (3-5). There is approximately 9-18% of ES in the head and neck exhibiting distant metastasis at the time of diagnosis, whereas this value is 0% in sinonasal region. The lungs and skeleton are the most common metastatic regions (5). As ES and peripheral PNETs share the same pathological entity, the World Health Organization refers them as ES/PNET. The diagnosis of ES requires strong positivity for CD99 (Fig.4), and/ or synaptophysin or chromogranin. 

**Fig 5:**
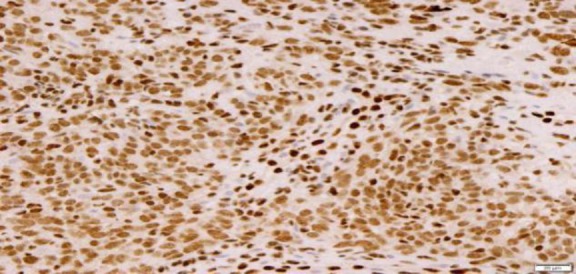
Immunohistochemical stain showing positive results for FLI1

The cytoplasm of tumor cells frequently contains perioidic acid Schiff-positive glycogen. Hallmark translocation of ES involving the fusion of the ES gene on chromosome 22 with the friend leukaemia virus integration site 1 (FLI1) gene on chromosome 11 shows the characteristic translocation t(11;22) (q24;q12) which is present in 85% and > 90% of PNETs/ES and extraosseous ES, respectively (Fig.5) (6). It can be revealed by using fluorescence in situ hybridization or polymerase chain reaction techniques (7).

There are various differential diagnoses for small round blue cell tumors (SRBCTs), such as epithelial tumors (including poorly differentiated squamous cell carcinoma), mesenchymal tumors (including rhabdomyosarcoma), lymphoproliferative disorder (including lymphoma), and neuroendocrine/ neuroectodermal tumors (including malignant melanoma and olfactory neuroblastoma). The extra panel of immunohistochemical stains and markers will be performed in order to establish final diagnosis and precise treatment. They include cytokeratin and anti-endomysial antibody (epithelial markers), Desmin, myogenin (rhabdomyosarcoma), CD3, CD20, CD45, Tdt (lymphoma including lymphoblastic lymphoma markers), HMB 45, S100 protein, Melan-A (melanoma), neuron-specific enolase, chromogranin A, synaptophysin, and CD56 (neuroendocrine/ neuroectodermal differentiation) (8-9). In this patient, the malignant cells were strongly positive for FLI1, CD99, and BCL and were focally positive for CD56 and synaptophysin. In the absence of other mentioned differential markers for SRBCTs, a final diagnosis of ES was made. Medical imaging, such as CT or magnetic resonance imaging (MRI), are usually performed to evaluate the extent of local disease and metastasis. The common CT findings in ES are usually expansile, moth-eaten permeative bony destruction, associated with soft tissue components without calcification. Periosteal reaction is usually aggressive in nature presenting either lamellated (onion-peel) or spiculated (sunburst or hair-on-end). The MRI features included reactive sclerosis pattern with hypointense to isointense on T1W1 and hypointense to hyperintense on T2W2. Moreover, the areas of hemorrhage and necrosis can be observed in MRI (10,11). Some studies even suggested further metastatic workup, such as Technetium-99m scintigraphy, bone scan, and bone marrow biopsy (12,13). In the present case, only CT scan was performed for disease staging. The CT findings showed a localized disease without any distant metastasis; thus, neither MRI nor bone scan was performed. Furthermore, in our center these scans were not easily accessible and will further delay on commencing treatment. The ES is known to be both chemosensitive and radiosensitive. Therefore, it is usually treated with multi-modal approaches. The main idea is to treat the local disease, followed by systemic therapy to eradicate micro or distant metastasis. The majority of centers use three- to four-drug chemotherapy regimen consisting of a combination of such agents as vincristine, doxorubicin, cyclophosphamide, ifosfamide, etoposide, adriamycin, actinomycin D, and/or cisplatinum. 

The choice of local treatment depends on the size and resectability of the tumor, as well as the primary site and critical surrounding structures with possible complications. Some prefer radiotherapy to surgery, due to difficulties in obtaining clear surgical margins, as well as the possible disfigurement and destruction caused by surgery (13). Siegal et al. outlined that patients with biopsy alone or complete surgical resection had greater survival rates, compared to incomplete excision. Raney et al. reported that patients with complete tumor removal prior to chemotherapy had greater chance of survival. However, there is limitation for obtaining negative margin of the sinonasal tract (14). In our patient, endoscopic surgical resection was opted, followed by chemotherapy. The given VIDE regimen was one of the standard regimens used globally (15). Since the surgical margin was clear as per EE99 protocol (15), radiation to the primary site was not planned. 

Our patient was subjected to endoscopic medial maxillectomy with modified Denker’s procedure. In this regard, he underwent endoscopic transnasal inferior turbinectomy and uncinectomy, followed by wide middle meatal antrostomy performed in the usual manner. Subsequently, incision was made inferiorly at the junction of nasal floor and lateral nasal wall (down to the periosteum), and superiorly along the lateral nasal wall extending to anterior-inferiorly up to the anterior end of inferior turbinate, overlying the edge of pyriform aperture. Subperiosteal flap was raised with a freer suction to expose the anterior aspect of the maxilla up to the infraorbital foramen and lateral nasal wall. 

Osteotomy was performed at the anterior wall of maxilla and connected to the inferior bony cut of medial maxillectomy. The osteotomy was bounded superiorly by the roof of the maxillary sinus, inferiorly by the junction of nasal floor and medial maxillary wall, and posteriorly by the posterior wall of maxillary, preserving the infra-orbital neurovascular bundle. Thereafter, the lesion was resected and removed en bloc. Nasolacrimal duct was identified and marsupialized with sickle knife to prevent stenosis.

**Table 1:** Summary of reported extraskeletal Ewing sarcoma in sinonasal tract

**Study (year)**	**Cases**	**Age (mean) and gender**	**Site**	**Presentation**	**Treatment given**	**Mets**	**Local recurrence**
Pontius & Sebek (16) (1981)	1 case	39YO, M	Nasal cavity and paranasal sinus	Epistaxis, nasal obstruction, malar pain, epiphora	Surgery and post-op RT	Nil	Nil
Siegal et al. (1987)	29 cases	10.9 YO , M:F = 1.23	Skull (38%), cervical vertebrae (24%), mandibular (21%), maxilla (14%), ethmoid sinus (3%),	Mass (48%), central nervous system and ocular effects (38%), swelling at the site of tumour (17%),	CHT + RT + biopsy or complete resection (76%), CHT + RT + incomplete resection (24%)	Nil	Nil
Lane et al. (1990)	1 case	7YO, M	Nasal cavity with ethmoid sinus	Eye swelling with diplopia	Surgery with post-op CHT	N/A	N/A
Allam et al. (1999)	24 cases	16.5 YO (median), M:F = 2.4	Maxilla (37.5%), mandible (25%), orbit (17%), skull (12.5%), nasal cavity (8%)	Painful swelling (90%)	Initial biopsy + combined CHT + RT (58%), surgery + post-op CHT + RT (21%), surgery + post-op RT (8%), surgery alone (8%)	Metastatic at diagnosis (12.5%), distant metastasis (46%): lungs (27%)	29%
Mark et al. (2003)	1 case	14YO, F	Ethmoid sinus	Nasal symptoms (purulent discharge, nasal obstruction, epistaxis)	Surgery + post-op CHT + RT	Nil	Nil
Caner et al. (2005)	1 case	14YO, M	Paranasal sinus (maxillary, sphenoid, ethmoid) extending left orbit and middle cranial fossa	Cheek swelling, nasal obstruction, headache	CHT + RT	N/A	N/A
Saurabh et al. (2007)	1 case	15 YO, M	Maxilla with intraorbital extension	Nasal symptoms (obstruction, discharge, epistaxis), painful facial swelling, ocular symptoms (vision impaired, epiphora and proptosis)	Operation with post-op CHT + RT	N/A	N/A
Sara Hafezi et al. (2010)	14 cases	32.4 YO, M:F = 0.56	Nasal cavity (36%), one or more sinuses (36%), both nasal cavity and at least one sinus (28%). Involved sinus: maxillary (36%), ethmoid (36%), sphenoid (14%) and frontal (14%).	Nasal obstruction and/or epistaxis	Combined CHT + RT (21%), surgery alone (14%), CHT alone (7%), surgery with post-op RT (7%)	Breast mets (7%), lung mets (7%)	14% dead of local disease (N/A for local recurrence date)
Dutta et al. (2014)	1 case	67 YO, M	Maxillary sinus	Painful swelling over left cheek	Surgery + post-op CHT + RT	Nil	Nil
Bivas et al. (2015)	35 cases	12 YO (median), M:F = 2.5	Maxilla & maxillary sinus (40%), mandible (20%), orbit (15%)	Swelling (94%), pain (37%), systemic symptoms (14%)	Combined CHT + RT (66%), CHT + surgery + post-op RT (23%), CHT alone (8%), CHT + surgery (3%)	Lung (3%), bone (3%), bone marrow (3%)	9%
Maria et al. (2015)	1 case	33 YO, M	Sinonasal tract with ethmoid/sphenoid sinus involvement and intracranial extension	Anosmia, epistaxis, reduction of visual acuity, headache	Surgery with post-op CHT + RT	Nil	Nil
Firas et al. (2015)	1 case	22 YO, F	Maxillary sinus	Cheek swelling with pain	CHT + RT	Nil	Nil
Davide et al. (17 ) (2016)	5 cases	36 YO (median), M:F=0.2	Nasoethmoidal complex (80%), maxillary antrum (20%)	Nasal obstruction (60%), epistaxis (60%), diplopia (20%), headache (20%)	CHT + RT + surgery (80%), combined CHT + RT (20%)	Sacrum (20%), leptomeningeal (20%)	20%
Tomoharu Suzuki et al. (18) (2017)	1 case	23YO, M	Nasal cavity, maxillary antrum and ethmoid sinus	Purulent rhinorrhea, nasal obstruction, and epistaxis	Surgery and post-op CHT + RT	Nil	Nil

YO: years old, Mets: metastasis, M/F: male to female ratio, CHT: chemotherapy, RT: radiotherapy, +/-: with or without, post-op: post operative, N/AL: not applicable

An analysis conducted by Bivas et al. showed that baseline high white blood cell count (>11,000/μL) was an independent predictor of the worst event-free survival, due to possible micrometastatic disease without overt metastasis. Histologic filigree pattern carried poorer prognosis, based on the evaluation of the Intergroup Ewing Sarcoma Study (IESS) data in 1983 (19). Several studies revealed that the main prognostic factor affecting the patients’ overall survival and disease-free survival is the response to chemotherapy treatment. Local control rate of the disease is attributed to the initial tumor size (large if >10 cm) and total delivered radiation dose (suggested median dose: 5040 cGy). Local recurrence rate was up to 29% in the studies carried out by Allam et al. In addition, metastatic spread at presentation with marked tumor necrosis is considered to have inferior outcome (20).According to the IESS, primary ES in the head and neck is proven to have better prognosis and lower mortality, compared to that in other anatomic locations. Nonetheless, the ES of the head and neck needs particular attention due to its proximity to vital structures, such as the orbit, brain, and major neck vessels, especially in case of local treatment, either in the form of surgery or radiotherapy. Therefore, therapy should be individualized, depending on the site of involvement with adequate reconstructive surgery to prevent further mutilation, morbidity, and mortality if it is considered likely that the benefits outweigh the risks.

## Conclusion

The ES is extremely rare to occur as a primary tumor in the head and neck region, especially at PNS; however, it has better prognosis and lower mortality. Early and accurate diagnosis, as well as aggressive intervention with multimodality approaches, are crucial to obtain good prognosis and functionality after the treatment. However, patient still needs to be followed up closely since local recurrence and distant metastasis are common.
